# Numerical investigation of aerosol transport in a classroom with relevance to COVID-19

**DOI:** 10.1063/5.0029118

**Published:** 2020-10-01

**Authors:** Mohamed Abuhegazy, Khaled Talaat, Osman Anderoglu, Svetlana V. Poroseva

**Affiliations:** 1 Mechanical Engineering Department, University of New Mexico, Albuquerque, New Mexico 87106, USA; 2 Nuclear Engineering Department, University of New Mexico, Albuquerque, New Mexico 87106, USA

## Abstract

The present study investigates aerosol transport and surface deposition in a realistic classroom environment using computational fluid-particle dynamics simulations. Effects of particle size, aerosol source location, glass barriers, and windows are explored. While aerosol transport in air exhibits some stochasticity, it is found that a significant fraction (24%–50%) of particles smaller than 15 *µ*m exit the system within 15 min through the air conditioning system. Particles larger than 20 *µ*m almost entirely deposit on the ground, desks, and nearby surfaces in the room. Source location strongly influences the trajectory and deposition distribution of the exhaled aerosol particles and affects the effectiveness of mitigation measures such as glass barriers. Glass barriers are found to reduce the aerosol transmission of 1 *µ*m particles from the source individual to others separated by at least 2.4 m by ∼92%. By opening windows, the particle exit fraction can be increased by ∼38% compared to the case with closed windows and reduces aerosol deposition on people in the room. On average, ∼69% of 1 *µ*m particles exit the system when the windows are open.

## I. INTRODUCTION

Transmission of COVID-19 occurs primarily through SARS-CoV2-laden droplets and aerosol particles inhaled directly or transmitted from contaminated surfaces.^1^ Effective mitigation measures necessitate clear understanding of droplet and aerosol transport, surface retention, and evaporation kinetics in different environments and conditions.^2^ Aerosols are generated during exhalation, talking, coughing, sneezing, and other activities.^3,4^ In indoor environments, some of the generated particles exit the system through ventilation, some deposit on surfaces in the room and may settle or re-enter the air, and others may be directly inhaled. Of primary interest to mitigation measures is maximizing the fraction of particles that exit the system and minimizing aerosol deposition on people to reduce disease transmission.^5,6^

Aerosol transport within a control volume is primarily affected by inertial forces due to airflow and drag on the particle, and gravitational sedimentation.^7^ The forces acting on a particle primarily depend on particle size and its position in the flow field. For smaller particles (<0.5 *µ*m), Brownian force can play a significant role in aerosol transport but becomes less important with increased particle size.^7,8^ The velocity field of the fluid (air) under known boundary conditions can in principle be estimated by numerically solving Navier–Stokes equations through direct numerical simulations (DNS), or more practically by numerically solving Reynolds-Averaged Navier–Stokes (RANS) equations with approximate turbulence closures such as k-ϵ and k-ω closures.^9,10^

As particle properties significantly affect aerosol and droplet transport within a system, it is necessary to consider accurate particle shape, size, and evaporation kinetics. The distinction between aerosols and droplets is rather arbitrary with no general agreement on a particle size threshold or suspension time threshold.^3^ However, droplets are typically considered to be larger particles where evaporation kinetics is rapid leading to the production of smaller aerosols with slow evaporation kinetics.^3^ Aerosol particles and droplets released from activities such as exhalation, talking, or coughing are polydisperse in nature. Exhalation and talking release particles mostly <1 *µ*m,^11^ and coughing releases larger particles typically <10 *µ*m,^12^ while sneezing was found in one study to release particles characterized by a bimodal size distribution with peaks of ∼386 *µ*m and 72 *µ*m and the corresponding geometric standard deviation of 1.8 and 1.5, respectively.^13^

Computational fluid dynamics has been used in many studies to investigate aerosol transport in outdoor conditions,^14^ indoor conditions such as hospitals,^6,15^ and even inside the human airway system with good agreement with the experimental data.^16,17^ During the COVID-19 pandemic, significant efforts have been made to develop computational fluid dynamics models of the human sneeze,^18^ investigate mask mechanics,^19^ and study aerosol transport and air flow in different environments and conditions such as aircrafts,^20^ vehicular cabins,^1^ urinals and toilets,^21,22^ public spaces,^23^ and indoor spaces.^24,25^ Despite these efforts, to the authors’ knowledge, no studies have investigated aerosol transport in a classroom environment although classroom sizes, the air conditioning layout, and aerosol source distribution are characteristically different than hospital care units and other indoor spaces discussed in the literature.

While a typical 900 sq. ft classroom can fit 18 students and an instructor, guidelines for re-opening schools have restricted the number of students to less than 10 students with 6 ft minimum spacing between the students. The effectiveness of these measures is dependent in part on aerosol transport within the classroom’s air conditioned environment, which remains under-characterized. Other strategies for COVID-19 mitigation may include the use of glass screens as barriers to reduce aerosol transport between people in the room, opening windows, and redistributing students in classrooms, but the ability of these measures to reduce aerosol transmission from one person to another needs to be carefully evaluated.

The objective of the present work is to investigate aerosol transport and surface deposition in a model classroom environment using computational fluid-particle dynamics (CFPD) simulations. Particularly, it is of interest to estimate the fraction of particles that exit the system, deposit on students, and deposit on surfaces such as desks, ground, walls, and ceiling. The effects of particle size, aerosol source location, glass barriers, and windows are investigated. Aerosol deposition on different students from different sources is compared to qualitatively explore the risk posed to individuals in the room due to their position with respect to an infected student.

## II. METHODS

### A. Classroom model and spatial mesh

**FIG. 1.**
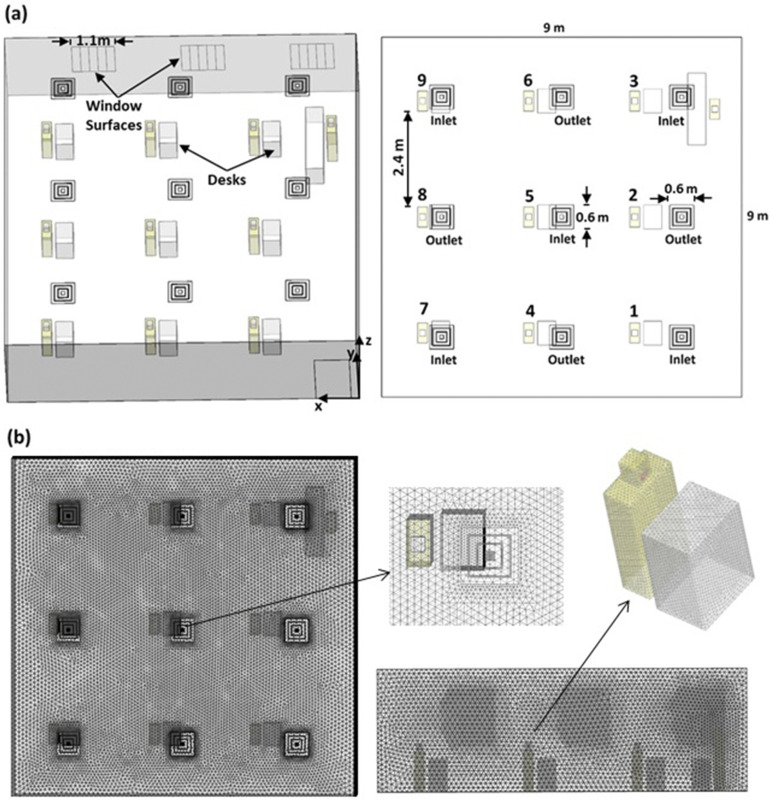
Illustration of (a) the classroom model and (b) the computational mesh used in the CFD simulations.

A three-dimensional model of a classroom consisting of nine students and an instructor was developed. The model uses realistic classroom dimensions and air conditioning. The classroom shown in Fig. 1 is 9 × 9 m^2^ in area and 3 m in height. The distance between each student is 2.4 m (7′ 10″), which is greater than the recommended 6 ft separation distance for COVID-19 mitigation. The model includes desks (with glass screens and without them) and windows. All students are represented similarly and have the same dimensions. Each student consists of a cuboid body (0.5 × 0.25 × 1 m^3^) and a cuboid head (0.16 × 0.15 × 0.2 m^3^) with a rectangular mouth surface (0.06 × 0.03 m^2^) through which particles and air are injected into the system. The simplified human model is inspired by models used in a numerical investigation of cross-transmission in hospitals.^6^ No chairs are considered in the model due to the extensive variability in chair sizes and shapes. Students are assumed to be exposed to aerosols in order not to underestimate deposition on students. An instructor is defined in the front, as shown in Fig. 1(a), and is assumed to be 1.7 m in height. Independent surfaces are defined in the model for each object for tracking the aerosol deposition on objects and students, respectively.

Air conditioning of the classroom follows ASHRAE 62.1 ventilation standards for acceptable indoor air quality.^26^ The air conditioning system consists of five supply diffusers and four return air diffusers distributed as shown in Fig. 1(a). The Cubic Feet per Minute (CFM) required for adequate ventilation was found to be ∼1230 CFM. The supply diffusers (1, 3, 5, 7, and 9) supply air at a 37° angle from the horizontal surface with an inlet flow area of 0.294 m^2^ and a diffuser inlet vertical air velocity of 0.395 m/s based on ASHRAE recommendations.^27^ In the present work, the effect of opening windows while the air conditioning system is running on particle removal is explored. For this purpose, the model includes 3 windows (2.2 × 1.3 m^2^) that can be opened up to 50% in 10% increments.

An unstructured, tetrahedral mesh is used, as shown in Fig. 1. The mesh was generated using ANSYS ICEM 19.1. The mesh consists of 3.3 × 10^6^ mesh cells with a minimum cell size of 0.5 cm and maximum cell size of 10 cm with gradual transition, maximum skewness of 0.823 (a mean value of 0.593), and maximum aspect ratio of 3.21 (a mean value of 1.43). The grid is refined near surfaces to maintain a wall y + <10 during the simulations. Each case of the 20 cases simulated in this work consumed ∼9 h running on four computer cores.

### B. Airflow and particle dynamics

The present study uses the commercial CFD code, ANSYS FLUENT 19.2, to simulate the airflow and particle transport. The continuity and momentum equations of the continuum phase (air) are solved in the beginning independent of the discrete phase using the steady state Reynolds Averaged Navier–Stokes (RANS) incompressible solver. The present simulations use the Re-Normalization Group (RNG) k-ε model.^28^ The choice of the RNG k-ε model is motivated by the work of Ramponi and Blocken who investigated the influence of turbulence models on cross-ventilation for a generic isolated building, and it was found that the RNG k-ε model was suitable for their application and operation conditions, which, in part, resemble the current application.^29^

The SIMPLE algorithm implemented in ANSYS FLUENT is applied for pressure velocity coupling with pressure interpolation of first order. The convection and viscous terms of the governing equations were discretized utilizing the second-order discretization scheme. The solution is assumed to be converged when all the scaled residuals stabilize and approach a minimum of 10^−5^ for k, ε, x, y, and z momentum equations as well as 10^−4^ for the continuity equation. Once the continuum phase solution converges, the flow field is then frozen and is used to transport the discrete phase (aerosol particles). The effect of the particles on the flow of air is negligible. One-way coupling between the continuum phase and the discrete phase is used given the low concentration of the aerosol particles in air. The particle trajectory is determined by solving the equation of motion for the particle in a Lagrangian framework. The equation of motion for the particles is given in the following equation [Eq. (1)]:

$$m\frac{d\vec{v_{i}}}{dt}=\vec{F_{drag}}+\vec{F_{g}}+\vec{F_{a}},$$ (1)

where v_i_ is the velocity of the particle, m is the mass of the particle, $\vec{F_{drag}}$ is the drag force between the air and the particle, $\vec{F_{g}}$ is the gravity force, and $\vec{F_{a}}$ represents the other additional forces including the pressure force, virtual mass force, Basset force, Brownian force, and Saffman’s lift force. The particles used in the present work are sufficiently small to neglect pressure and virtual mass forces and sufficiently large to neglect Brownian force.^7,8,30^ As the particles are much smaller than the mesh elements, it is necessary to use drag models. The present work uses the Stokes–Cunningham drag model. Therefore, the equation of motion of the particles could be written more explicitly as follows [Eq. (2)]:

$$\frac{dv_{i}}{dt}=\frac{f}{\tau_{p}C_{c}}\left(u_{i}-v_{i}\right)+g_{i}\left(1-\alpha \right)+f_{i,Brownian}+f_{i,lift},$$ (2)

where *u*_*i*_ is the velocity of the flow, *f* is the drag factor,^31^ *τ*_*p*_ is the particle reaction time, and *C*_*c*_ is the Cunningham correction factor.^32^ The present simulations use 96 000 particles, which is a reasonable number of particles for sound statistics and is greater than those used in another study of aerosol removal in hospital care units.^15^ The turbulent dispersion of particles and the random effects of turbulence on particle dispersion were taken into account using the discrete random walk method implemented in ANSYS FLUENT. Since the particles are small enough to stick to surfaces, the trap boundary condition is used for the particles over all solid surfaces. In reality, some of the particles will be reflected and others may re-enter the air after deposition. However, re-entry and reflection are difficult to account for as they are affected by particle properties, surface properties, and flow conditions.^33^ An escape boundary condition is employed for the diffusers and mouths. Air flow from mouths is assumed to be exhaled at 20 l/min specified as a velocity inlet boundary condition (0.185 m/s) for a mouth inlet area of 0.0018 m^2^. The particles are released with the same velocity normal to the mouth surface.

### C. Study design

The base case uses 1 *µ*m particles, student 5 as the source, no glass barriers, and windows closed. The choice of 1 *µ*m particles is in the range of the particle size of aerosol particles released in exhalation and talking.^11^ Student 5 is used as the source for the base case due to their location far away from vortices at the edges of the room.

The present study investigates the effects of particle size, source position, glass barriers, and windows on the fate of the exhaled aerosol particles. Parameters of the base case are varied to investigate these effects. Particle sizes studied are 1 *μ*m, 4 *μ*m, 10 *μ*m, 15 *μ*m, 20 *μ*m, and 50 *µ*m. Aerosol sources considered are students 1, 2, 5, 8, and 9 and are studied with and without 70 cm high glass barriers/screens placed on top of the desks. The effect of windows is explored by comparing aerosol deposition and transfer in the classroom with 0%, 10%, 20%, 30%, 40%, and 50% open windows. As three windows are available in the room, 10% open windows implies that 10% of each of the three windows is open. The effect of windows is explored while the air conditioning system is running. While this can increase the cooling/heating load and decrease the energy efficiency of the air conditioning system, the present work is concerned with the effect on particle removal. Table I summarizes the parameter combinations investigated in the present work.

**TABLE I.** List of parameter combinations investigated in the present work.

Investigation	Source	Particle size (*μ*m)	Screens	Windows
Effect of particle size	Student 5	1	No	Closed
	Student 5	4	No	Closed
	Student 5	10	No	Closed
	Student 5	15	No	Closed
	Student 5	20	No	Closed
	Student 5	50	No	Closed
Effect of source location	Student 1	1	No	Closed
	Student 2	1	No	Closed
	Student 5	1	No	Closed
	Student 8	1	No	Closed
	Student 9	1	No	Closed
Effect of glass barriers/screens	Student 1	1	Yes	Closed
	Student 2	1	Yes	Closed
	Student 5	1	Yes	Closed
	Student 8	1	Yes	Closed
	Student 9	1	Yes	Closed
Effect of windows	Student 5	1	No	10% open
	Student 5	1	No	20% open
	Student 5	1	No	30% open
	Student 5	1	No	40% open
	Student 5	1	N0	50% open

## III. RESULTS AND DISCUSSION

### A. Airflow and particle dynamics

**FIG. 2.**
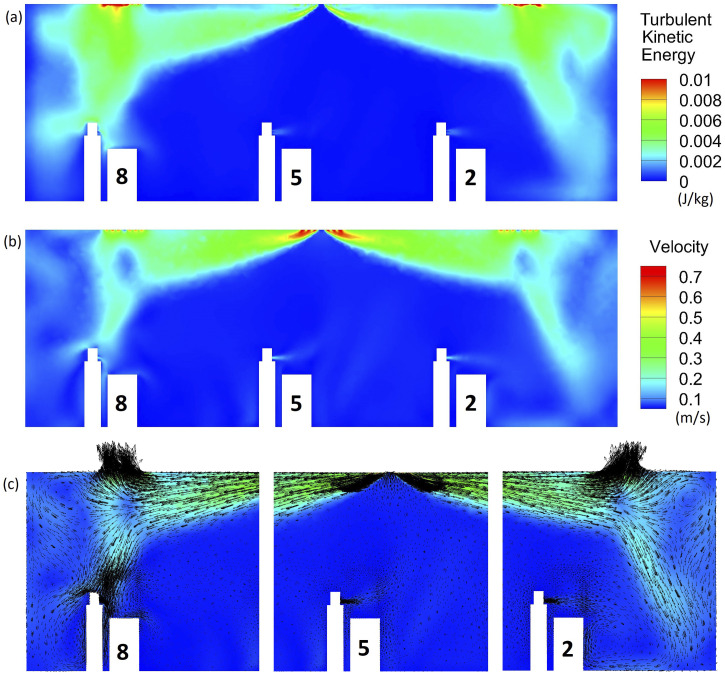
(a) Turbulent kinetic energy, (b) velocity magnitude distribution, and (c) velocity vectors across a slice going through students 2, 5, and 8.

The velocity field of the continuum phase and the distribution of turbulent kinetic energy and vorticity are of fundamental importance to aerosol transport. Figure 2 shows the turbulent kinetic energy distribution, velocity magnitude distribution, and velocity vectors of air across a two-dimensional slice going through students 2, 5, and 8. In this slice, air is injected into the system through the supply diffuser in the middle (inlet 5) at a 37° angle with the ceiling. Return diffusers 2 and 8 are shown at the sides. The turbulent kinetic energy is more significant at the edges of the room (especially at the outlets) and close to student 8 by the virtue of their location with respect to air conditioning [Fig. 2(a)]. The velocity magnitude is strongest at the inlets and outlets, but the air is not stagnant in the rest of the room due to air conditioning [Fig. 2(b)]. The velocity vectors [Fig. 2(c)] demonstrate the recirculation near the edges of the room and near student 8’s head. Vortices can partially trap aerosol particles that are transported to those regions and increase deposition on neighboring surfaces.

**FIG. 3.**
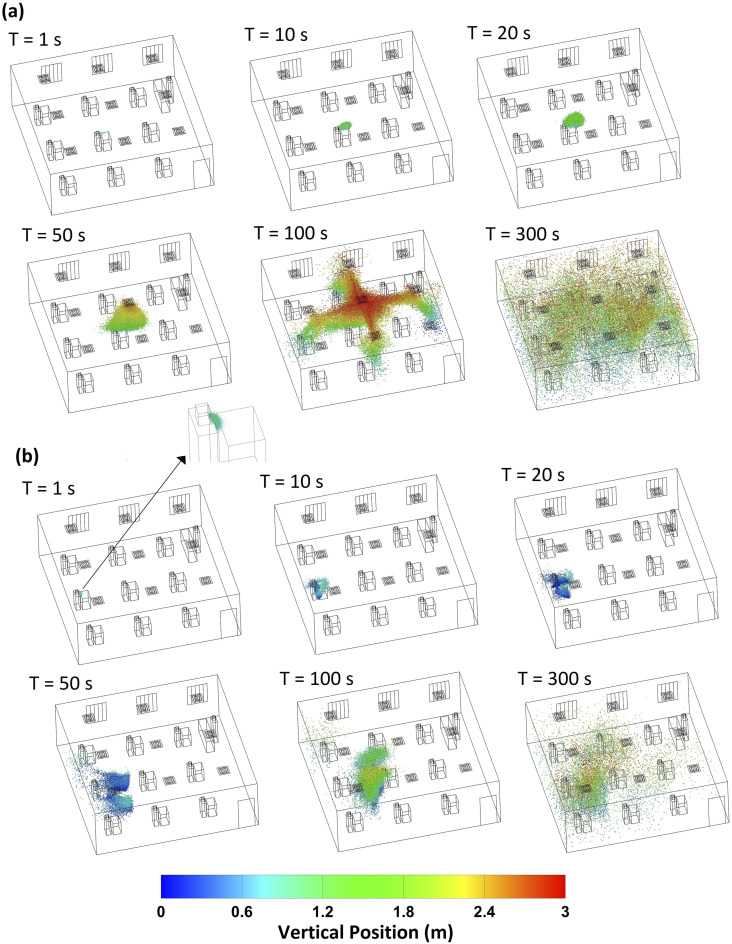
Distribution of 1 *µ*m aerosol particles in the classroom at different points in time for the (a) student 5 source and (b) student 8 source.

Particle transport in the classroom environment due to an impulse aerosol source is a transient process. For the purposes of characterizing the dynamics and the fate of exhaled aerosol particles, a single-release impulse source is used. Figure 3 shows the distribution of 1 *µ*m aerosol particles in the classroom at different points in time since particle release. Figure 3(a) illustrates the transport of particles released from student 5. After 1 s of release, the aerosol particles exhibit a parabolic distribution at the front of the particle swarm. The particles slowly disperse and rise up during the first 50 s. Once the particles reach the downstream of the air conditioner, the particles are rapidly transported to different parts of the room. As air flows from the supply diffusers to the return diffusers, the particles that reach the downstream of the air tend to follow the flow and exit the system. Overall, there are significantly more 1 *µ*m particles in the upper half of the room than the bottom half due to the flow of air to the return diffusers that are located in the ceiling in the present model. Figure 3(a) highlights the significance of the flow velocity distribution on aerosol transport in the room. Therefore, the results of the present work are applicable to classrooms with comparable air conditioning.

Figure 3(b) illustrates the transport of 1 *µ*m particles released from student 8. Aerosols released from student 8 [Fig. 3(b)] exhibit a substantially different distribution than aerosols released from student 5 [Fig. 3(a)]. At 1 s, the particle swarm curves downward and much of the particles deposit on the source student (student 8). This is a result of the position of student 8 with respect to the air conditioning system. As shown in Fig. 2, the velocity magnitude near student 8 due to air conditioning is strong compared to that near student 5. Student 8 is also present near a region with recirculation and strong vortices compared to the rest of the room. The particles disperse slowly, and even after 5 min, most of the particles are present in the back half of the room.

### B. Effect of particle size

**FIG. 4.**
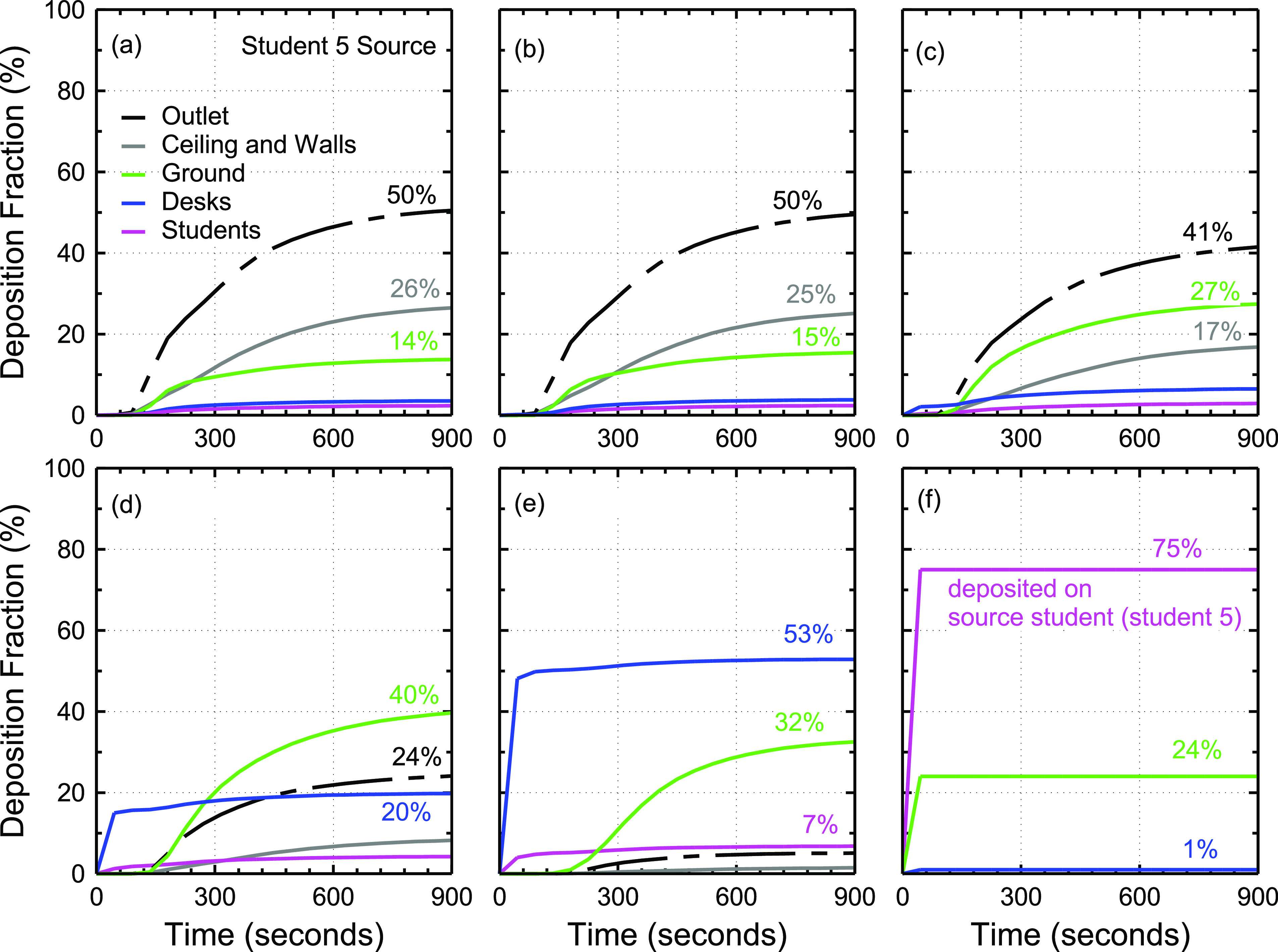
Effect of particle size on aerosol deposition and removal from the classroom model as a function of time since particle release from student 5’s mouth. This figure shows the deposition fraction for (a) 1 *µ*m particles, (b) 4 *µ*m particles, (c) 10 *µ*m particles, (d) 15 *µ*m particles, (e) 20 *µ*m particles, and (f) 50 *µ*m particles.

Particle size is of fundamental importance to aerosol transport.^7^ The present work considers spherical aerosol particles in the 1 *µ*m–50 *µ*m size range. Figure 4 shows the effect of particle size on the fraction of aerosol particles released from student 5’s mouth that deposit on different surfaces in the room, such as ground, ceiling and walls, desks, and students, or escape from the outlet of the air conditioning system. No significant difference is observed between 1 *µ*m and 4 *µ*m particles [Figs. 4(a) and 4(b)]. Nearly 50% of 1 *µ*m and 4 *µ*m aerosol particles exit the room through the air conditioning system after 15 min. Roughly 15% of the particles deposit on the ceiling, and ∼10% deposit on the walls of the classroom, which is comparable to 14%–15% deposition on the ground [Figs. 4(a) and 4(b)]. This suggests that gravity does not play a significant role in the transport of 1 *µ*m and 4 *µ*m particles in the timescale of 15 min. In the case of 10 *µ*m particles [Fig. 4(c)], deposition on the ground increases to 27% compared to 14%–15% in 1 *µ*m and 4 *µ*m particles [Figs. 4(a) and 4(b)] and the fraction of particles that exit the system through air conditioning is reduced to 41%. The fraction of aerosol particles that exit the system drops rapidly with the particle size greater than 10 *µ*m from 41% at 10 *µ*m [Fig. 4(c)] to 24% at 15 *µ*m [Figs. 4(d)], 16% at 17 *µ*m (not shown), 5% at 20 *µ*m [Figs. 4(e)], and 0% at 50 *µ*m [Fig. 4(f)]. On the other hand, the total fraction of particles that deposit on the ground, desks, and the source student increases significantly with increased particle size [Figs. 4(a)–4(f)]. For instance, ∼21% of 1 *μ*m particles deposit on the ground, desks, and students, while ∼92% of 20 *µ*m particles deposit on the ground, desks, and students. The rest of the particles deposit on the ceiling and walls, exit the room through the air conditioning system, or remain in the air for longer than 15 min.

Figure 4 also shows that ∼15 min is adequate for 1 *µ*m–50 *µ*m particles to have at least one interaction with a surface or exit the room. In the case of 50 *µ*m particles, the particles deposit rapidly in less than a minute and mostly on the source student. The extensive deposition of 50 *µ*m particles on the source student is due to gravitational settling and the simplified, rectangular geometry of the student modeled (Figs. 1 and 2). Much of these 50 *µ*m particles would deposit on the ground if not for the simplified student geometry.

### C. Effect of source location

**FIG. 5.**
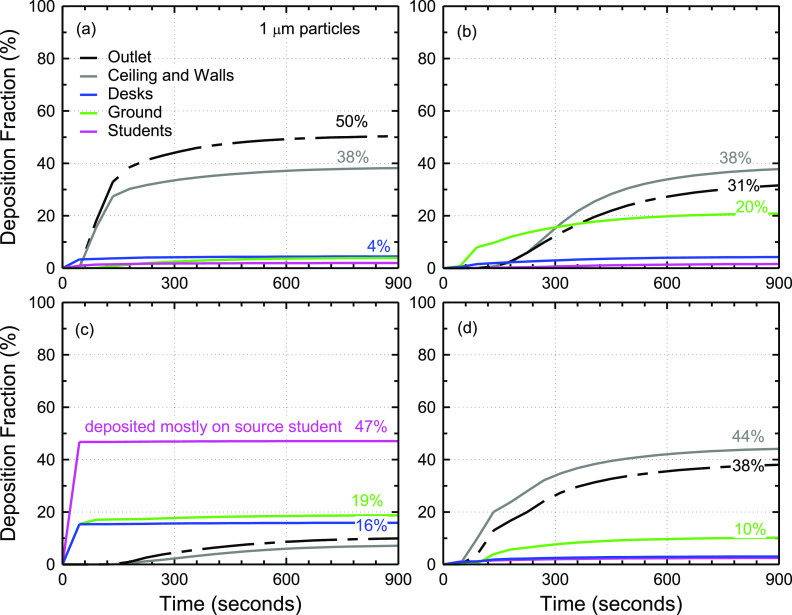
Effect of student location on aerosol deposition and removal from the classroom model using 1 *µ*m particles. This figure shows four different student sources: (a) student 1, (b) student 2, (c) student 8, and (d) student 9.

The position of the initial aerosol source in the fluid flow field affects the trajectory of the released particles [Eqs. (1) and (2)]. The location of the student with respect to air conditioning influences the local flow field and particle dynamics (Figs. 2 and 3). It is, therefore, of interest to understand the extent of the effect of source location on the fate of the exhaled particles. Figure 5 compares the aerosol deposition on various surfaces originating from different sources (students 1, 2, 8, and 9) using 1 *µ*m particles. The aerosol deposition for the student 5 source was shown earlier in Fig. 4(a).

The results in Fig. 5 show that the effect of source location on aerosol transport can be substantial as in the case for student 8. The deposition distribution in the case of student 1 [Fig. 5(a)] is similar to that of student 5 [Fig. 4(a)] except for very low aerosol deposition on the ground compared to student 5 (3.9% vs 13.7%) and the increased aerosol deposition on the walls and ceiling (∼38% vs 26%). The deposition results for student 9, who is positioned in the back corner, also suggest increased deposition on the wall and ceiling to ∼44% of exhaled aerosol particles. In the case of student 1 and student 9, the increased deposition on walls and ceiling can be explained in part by proximity to walls and in part due to the vortex structures present near the edges of the room (Fig. 2). Student 2 who is positioned in the front-middle, far from walls, experiences increased deposition on the walls and ceiling compared to student 5 [Figs. 5(b) and 4(a)]. This increase in deposition on the wall may be explained by the vortices present in the flow in front of student 2 (Fig. 2). The deposition on the ground appears somewhat stochastic due to the vortices, but in general, it is <20% for 1 *µ*m particles. The fraction of particles that exit through the air conditioning system is consistently >30% except for student 8 [Fig. 5(c)]. The case of student 8 is special due to their unique position with respect to the air conditioning system (Fig. 2), which directs the particles downward and onto themselves (Fig. 3). Less than 10% of the particles exhaled by student 8 exit the room through the air conditioning system.

### D. Effect of glass barriers/screens

One of the commonly used measures to reduce COVID-19 transmission is the use of sneeze guards in the form of glass or plastic barriers. The efficiency of barriers is not independent of the flow field where they are employed, which depends on air conditioning and the geometry of the surroundings. Therefore, it is necessary to evaluate its effectiveness in the classroom environment especially for small particles such as 1 *µ*m particles which can diffuse for long distances in the room.

**FIG. 6.**
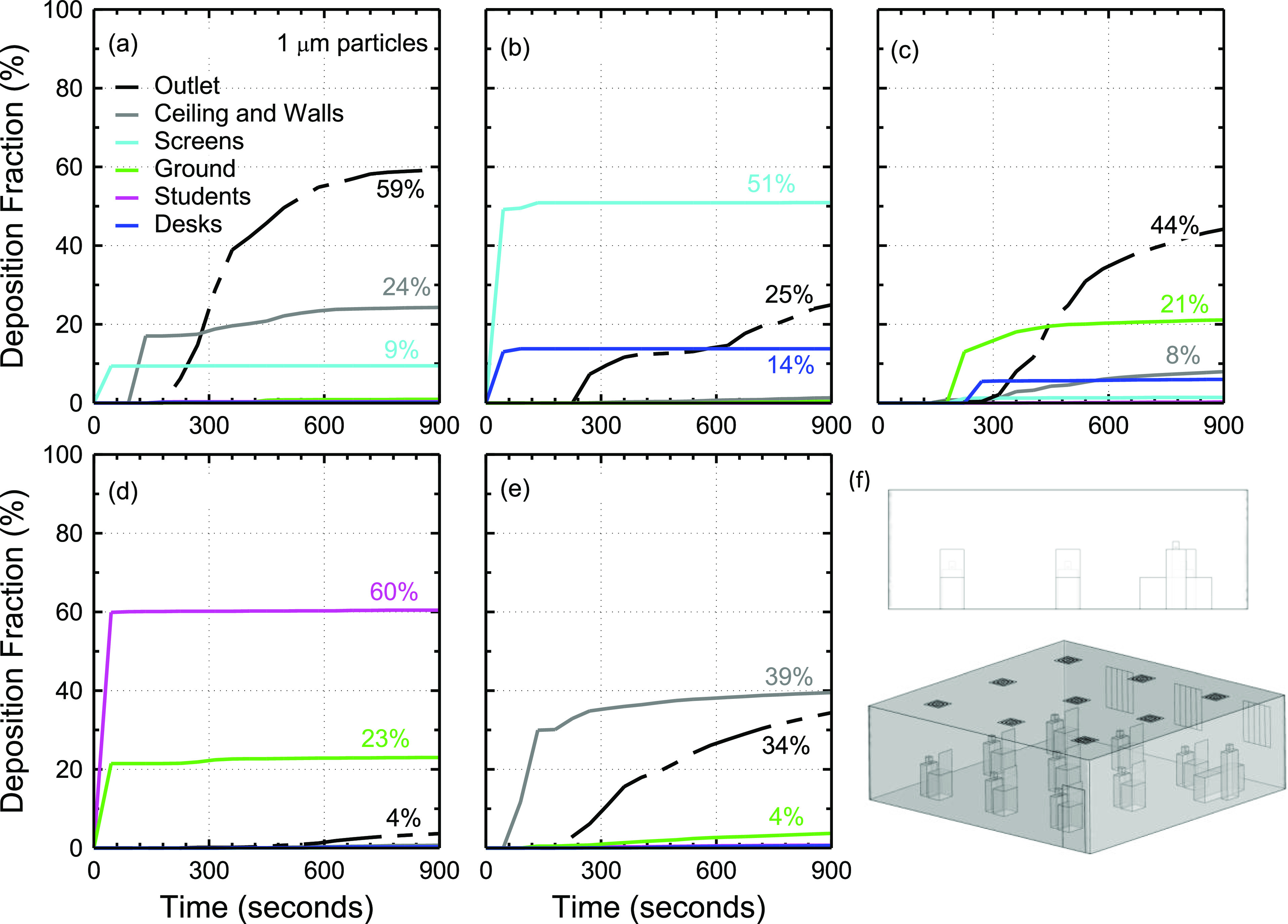
Effect of glass barriers on aerosol deposition and removal from the classroom model using 1 *µ*m particles for different student sources. This figure shows five different student sources: (a) student 1, (b) student 2, (c) student 5, (d) student 8, and (e) student 9. The glass barriers are shown in (f).

Figure 6 shows the deposition distribution of 1 *µ*m particles released from different student sources in the presence of 70 cm tall glass barriers on top of the desks [Fig. 6(f)]. The deposition of the particles on the screens varies significantly from one source to another. The fraction of 1 *µ*m particles deposited on the screens is very small (<1.5%) for students 5, 8, and 9 [Figs. 6(c)–6(e)]. More significant deposition on the screens is observed in the case of student 1 (9%) and student 2 (∼51%), as shown in Figs. 6(a) and 6(b). Differences in the aerosol deposition compared to the case with no barriers (Fig. 5) are also observed. The differences can be attributed to the modulation of the local flow field as a result of the barriers, which further depends on the position of the barrier in the flow field. Notably, the inclusion of barriers decreases the total fraction of particles deposited on the students by ∼63% on average compared to the case with no barriers. However, barriers appear to slow down aerosol removal and deposition. For instance, ∼20% of the particles remain in the air after 15 min in the case of student 9 when barriers are used, while only ∼3% of particles remain in the case with no barriers.

**FIG. 7.**
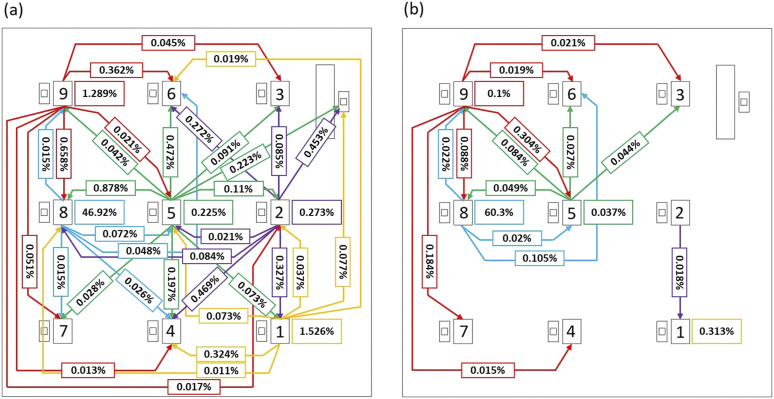
Effect of glass barriers on aerosol transmission between students. Sources considered are student 1, student 2, student 5, student 8, and student 9, and particle size is 1 *µ*m. Source–receiver maps are shown for cases with (a) no glass screens or sneeze guards and (b) glass screens employed.

It is difficult to assess the effectiveness of glass barriers in reducing aerosol transmission based on Figs. 5 and 6, which do not discriminate between the source student and receivers. For a clearer comparison, Fig. 7 shows source–receiver maps for 1 *µ*m particles in the absence and presence of screens. The sources considered are student 1, student 2, student 5, student 8, and student 9. Self-deposition is indicated in a box next to each student, and the fraction of aerosol deposited on other students is marked by arrows from the source to the receiver. A threshold of 0.01% (∼10 particles) is applied to the maps. The use of a threshold is to ensure that only statistically meaningful numbers are reported.

On average, the total fraction of aerosols transmitted from a source student to others in the classroom decreases by ∼92% in the case with screens. In the presence of screens, very few aerosol particles (<0.01%) are transmitted from student 1 to the others in the room, and self-deposition is significantly reduced from 1.5% to 0.3%. In the cases of student 2 and student 5, aerosol transmission to others and self is consistently reduced with the exception of increased transmission from student 5 to student 9 (from 0.04% to 0.08%). In the case of student 8, self-deposition increases from ∼47% to ∼60% and deposition on students 6 and 9 increases, but deposition on others decreases significantly. In the case of student 9, total aerosol transmitted to others is reduced by ∼74%. However, transmission from student 9 to student 5 increases from 0.02% to 0.3% and that from student 9 to student 7 increases from 0.05% to 0.18%. Overall, the addition of screens substantially reduces aerosol transmission from one student to another, but it does not eliminate particle transmission between students.

### E. Effect of windows

**FIG. 8.**
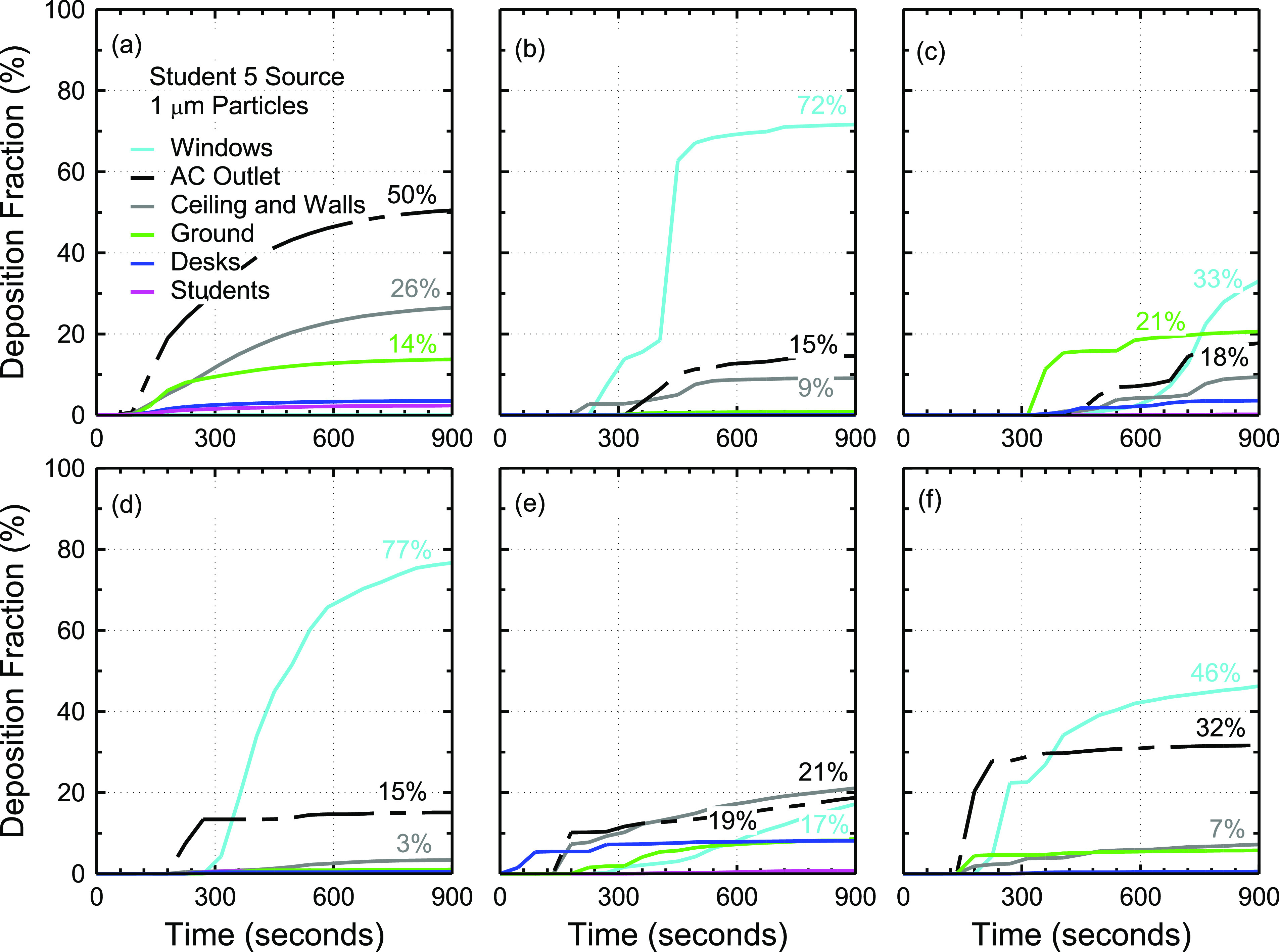
Effect of opening windows on aerosol deposition and removal using 1 *µ*m particles and the student 5 source. This figure shows the deposition fractions for cases with (a) 0%, (b) 10%, (c) 20%, (d) 30%, (e) 40%, and (f) 50% open windows.

The effect of opening windows while the air conditioning system is running is investigated in order to understand its impact on particle removal compared to the case with windows closed. A typical sliding window can be opened up to 50% of its total width. The present work considered cases with 0%, 10%, 20%, 30%, 40%, and 50% open windows using 1 *µ*m particles. The source student is assumed to be student 5. Figure 8 shows the effect of opening windows on aerosol deposition and removal.

The total fraction of particles that exit the system through the windows and air conditioning outlet is increased on average by ∼38% [Figs. 8(a)–8(f)]. The fraction of particles that exit the system through air conditioning is reduced by ∼60%. This is advantageous as fewer particles may be able to transfer to other rooms bypassing the air conditioning filters. The fraction of particles that exit through the windows appears to be affected by the extent to which the windows are open. The results shown in Figs. 8(a)–8(f) suggest that there may be an optimal configuration such that the fraction of particles that exit the system is maximized although no systematic trend is observed. The fraction of particles that exit the system for 0%, 10%, 20%, 30%, 40%, and 50% open windows is 50%, 87%, 51%, 92%, 36%, and 78%, respectively. On average, ∼69% of particles exit the system when windows are open at all, compared to 50% with windows closed. With the exception of 40% open windows, opening windows increases the fraction of particles that exit the system.

### F. Discussion

The results demonstrate that a large fraction (24%–50%) of smaller particles (<15 *µ*m) exit the room without interacting with any surfaces in the room. This finding highlights the need for efficient filtering in the air conditioning systems. The aerosol released from students disperses in the room, and its concentration decreases. The concentration of the aerosol particles increases again as they enter the air conditioning system. The transfer of a larger fraction of exhaled particles to the air conditioning return diffuser, although beneficial to individuals in the room, may pose greater risk to individuals in other rooms as air conditioning systems often use recycled air. It is also found that a 2.4 m separation distance between students is inadequate to eliminate particle transmission between students with the exception of 50 *µ*m particles.

The fraction of particles that exit the system without interacting with any surfaces depends on the source location. Interestingly, students closer to the supply diffusers such as student 1, student 5, and student 9 are associated with greater particle exit fractions than students closer to outlets such as student 2 and student 8. The position of the student in the flow field significantly affects particle transport. Significant aerosol deposition (∼47%) on student 8 is observed due to the aerosol they released. This is due to their unique position in the flow field near a vortex region close to the edge of the room and close to an outlet. An important implication of this increased aerosol deposition on student 8 is that it suggests the presence of mixing hotspots in the room where aerosol deposition can increase by as much as tenfold. In such a hotspot, if two students are present, the chances of aerosol transmission between the two will be significantly higher than elsewhere in the room. This highlights the need for thorough characterization of aerosol transport in different environments to identify and avoid hotspot areas.

Sneeze guards/glass barriers were found to effectively reduce the transmission of 1 *µ*m aerosol between students by ∼92% on average. While the fraction of particles deposited on the screens directly is small in most cases studied, the screens appear to modulate the local flow field resulting in less aerosol transmission between students. Screens, however, do not completely eliminate transmission of 1 *µ*m particles between students and their effectiveness depends on source location within the classroom with respect to the air conditioning system. Nevertheless, the 92% reduction in aerosol transmission is highly beneficial.

Opening windows was found to increase the fraction of particles that exit the system by ∼38% compared to the case with closed windows. The fraction of aerosol particles that deposit on students (including the source) decreased from 2.3% to an average of 0.45% when windows are open at all suggesting that opening windows reduces aerosol deposition on students by ∼80%. The present study only investigated one source (student 5) for cases with open windows. However, the results suggest that opening windows while the air conditioning system is running reduces aerosol transmission between students and increases the fraction of particles that exit the system.

The present work is subject to many limitations. First, deposition of aerosol particles on contact with solid surfaces is assumed. Reflection and re-entry are not considered. This is, however, justified as most of the simulations conducted in this study are of 1 *µ*m particles. Particles <50 *µ*m in diameter can stick to surfaces through van der Waals forces.^34^ Adhesion forces acting on 1 *µ*m particles can exceed gravitational force acting on the particle by factors greater than 1 × 10^6^.^34^ Adhesion forces, however, depend on particle properties, surface properties, and environmental factors.^33^ Second, the present work does not investigate the synergy between the different factors considered. For instance, the effect of opening windows on aerosol removal and deposition is not necessarily independent of particle size. Nevertheless, investigating the synergy between the different variables would necessitate extensive computational resources not available to the current project. The current study is rather focused on identifying what factors are important for aerosol transport in a classroom in order to inform other studies that may further investigate the interactions between the different factors. Third, the deposition fraction is assumed to be a single deterministic value. Statistical characterization of the deposition fraction would be of interest especially because of the existence of recirculation and vortices near the edges of the classroom. Fourth, classrooms are subject to extensive variability in sizes, air conditioning, student distribution, and student age/, which would affect aerosol deposition and removal. Effective mitigation strategies should consider multi-layer approaches including using masks, redistributing students, using glass barriers, opening windows, optimizing the air conditioning system for maximum particle removal, and improving air conditioning filters.

## IV. CONCLUSIONS AND RECOMMENDATIONS

Understanding aerosol transport in different environments is of critical importance to COVID-19 mitigation measures. The present study investigated aerosol removal and surface deposition in a realistic classroom environment using computational fluid-particle dynamics (CFPD) simulations. A model classroom that included nine students and a teacher was constructed. Air conditioning of the classroom followed ASHRAE 62.1 ventilation standards for acceptable indoor air quality. Four different factors were considered: particle size (1 *µ*m–50 *µ*m), source location (students 1, 2, 5, 8, and 9), presence of barriers/sneeze guards, and opening windows (10%–50% of window width). The following points highlight the main findings of this work and the implications of these findings:

(a) Aerosol distribution in the room is not uniform and is strongly influenced by the air conditioning layout.
(b) Even with only 9 students in the room and 2.4 m distance between students, the aerosol (1 *µ*m–20 *µ*m) is transmitted in significant quantities between students and from one student to other students’ desks with aerosol transmission between two neighboring students reaching 0.9% of exhaled particles in some 1 *µ*m particle cases. Studies have estimated that ∼20 000 particles in the 0.8 *µ*m–5.5 *µ*m range are released and that over 100 000 virions are emitted per minute of speaking.^35,36^ Therefore, particles transmitted between neighboring students separated by a 2.4 m distance in a classroom may exceed 180 particles per minute. The transmission of particles from one student to other students’ desks highlights the need for hand sanitization even without contact with other students’ belongings.
(c) The effect of source location on aerosol transport is significant. Student 1 in the front corner transmitted ∼0.55% of exhaled 1 *µ*m aerosol particles to other students, while student 5 in the middle transmitted ∼2.1% of exhaled particles to others. Removing the middle student seat (student 5) may help reduce the risk of infection to others. Furthermore, student position appears to affect the likelihood of receiving aerosol particles from others. Students 7 and 9 in the back corners received 2 to 3 times less particles on average than most other students in the room. Therefore, students at risk of COVID-19 complications may be placed in positions with a lower chance of receiving particles.
(d) Opening windows while the air conditioning system is running, while not recommended from an HVAC point of view, significantly increases particle exit fraction by ∼38% and reduces transmission between students by ∼80%.
(e) Glass screens reduce aerosol transmission from one student to another and should be used. The extent of their effectiveness depends on the source location with respect to the air conditioning system.
(f) Particles disperse in the room and re-concentrate at the return ducts of the air conditioning system. A large fraction of exhaled particles end up in the air conditioning system, which highlights the need for effective filtration and sterilization systems within air conditioners.

Finally, the results of this work should be interpreted under the context of the air conditioning layout and student distribution used. Other classrooms may employ different air conditioning standards and might necessitate aerosol transport investigations tailored to the specific classroom. Each case of the 20 cases simulated in this work consumed ∼9 h running on four computer cores. Notably, this runtime was enabled by freezing the continuum solver upon convergence of the residuals. Only the discrete phase transport is solved for a simulation time of 15 min.

## SUPPLEMENTARY MATERIAL

See the supplementary material for a high resolution (7832 × 3168 pixel^2^) figure showing the velocity vectors of Fig. 2(c) for greater clarity.

## AUTHORS’ CONTRIBUTIONS

M.A. and K.T. contributed equally to this work.

## Data Availability

The data that support the findings of this study are available from the corresponding author upon reasonable request.
